# Cytotoxic Phenolic Compounds from Fruit Glandular Trichomes of *Macaranga tanarius*

**DOI:** 10.1155/2019/2917032

**Published:** 2019-10-13

**Authors:** Thi Mai Huong Doan, Thuy Linh Nguyen, Thi Thanh Van Trinh, Van Nam Vu, Thi Dao Phi, Marc Litaudon, Fanny Roussi, Van Minh Chau, Van Cuong Pham

**Affiliations:** ^1^Advanced Center for Bioorganic Chemistry, Institute of Marine Biochemistry, Vietnam Academy of Science and Technology, 18 Hoang Quoc Viet, Caugiay, Hanoi, Vietnam; ^2^Graduate University of Science and Technology, VAST, 18 Hoang Quoc Viet, Caugiay, Hanoi, Vietnam; ^3^Institut de Chimie des Substances Naturelles, CNRS, ICSN UPR2301, University of Paris-Saclay, 91198 Gif-sur-Yvette, France

## Abstract

A new flavonoid, macatanarin D (**1**), together with five known stilbenes (**2-6**), was isolated from fruit glandular trichomes of *Macaranga tanarius*. Their structures were elucidated on the basis of spectroscopic methods and through comparison with data reported in the literature. All isolated compounds were evaluated for their cytotoxic activities against KB and MCF-7 cell lines. Compounds **3**, **4**, and **5** showed the strongest activities against both cell lines with IC_50_ values in the range of 0.03–0.12 *μ*M, and compound **2** only showed a significant cytotoxicity against KB cell line (IC_50_ = 0.26 *μ*M) and a moderate cytotoxicity against MCF-7 (IC_50_ = 10.4 *μ*M). Compounds **1** and **6** showed weak cytotoxic activities against KB cell line with IC_50_ values of 29.3 and 24.7 *μ*M, respectively.

## 1. Introduction

*Macaranga* (Euphorbiaceae) is a large genus of about 300 species mainly distributed in Southern Asia, of which 13 species are native to Vietnam [1, 2]. In Vietnam, several species of this genus known as “Ba soi” have been used in traditional medicine for the treatment of swellings, wounds, and diarrhoea [2, 3]. Phytochemical studies of *Macaranga* species have led to the discovery of various compounds such as flavonoids [4–6] and stilbenes [7, 8], which are regarded as the main constituents [9]. They are responsible for the cytotoxic and antioxidant activities generally found in plants of this genus [9]. *Macaranga tanarius* is known as “Bach dan nam” in Vietnam. The dried roots are used as an emetic agent, whereas fresh leaves are used as an anti-inflammatory drug to heal wounds [10].

A previous chemical investigation of *Macaranga tanarius* fruits led to the isolation of seven new and six known prenylated stilbenes [11]. In another study, it was also demonstrated that vedelianin, one of the most potent cytotoxic metabolites of this chemical series, was located in the glandular trichomes present on the surface of fruits of this species [12]. Plant glandular trichomes are considered to be natural cell factories of high biotechnological interest [13]. This result, combined with the highly cytotoxic activity of an AcOEt extract of glandular trichomes of *Macaranga tanarius* fruits, led us to further investigate chemically these epidermal outgrowths. Herein, we report the isolation of five known prenylated stilbenes (**2**–**6**) and the structure elucidation of the new flavonoid macatanarin D (**1**) and their cytotoxic activities against KB and MCF-7 cancer cell lines.

## 2. Materials and Methods

### 2.1. General Experimental Procedures

Optical rotations were determined on a JASCO P-2000 polarimeter (Hachioji, Tokyo, Japan). High-resolution ESIMS was measured on a Varian 910 spectrometer (Varian, California, USA). IR spectra were obtained on a Bruker 23 TENSOR 37 FT-IR spectrometer (Bruker, Billerica, MA, USA). UV spectra were measured using a UV-1601 spectrometer. The ^1^H and ^13^C, HMQC, HMBC, NOESY/ROESY, and COSY NMR spectra were recorded on a Bruker AM500 FTNMR spectrometer (Bruker, Billerica, MA, USA), and tetramethylsilane (TMS) was used as an internal standard. Column chromatography (CC) was performed using a silica gel (Kieselgel 60, 70–230 mesh and 230–400 mesh, Merck, Darmstadt, Germany) or Sephadex™ LH-20 (Supelco, Bellefonte, PA, USA). Thin-layer chromatography (TLC) used precoated silica gel 60 F254 (1.05554.0001, Merck, Darmstadt, Germany), and compounds were visualized by spraying with aqueous 10% H_2_SO_4_ and heating for 1.5–2 min.

### 2.2. Plant Samples

The fruits of *Macaranga tanarius* were collected in A Luoi, Thua Thien Hue, Vietnam, in June 2017 and were identified by Dr. Nguyen The Cuong of the Vietnam National Museum of Nature, Vietnam Academy of Science and Technology (VAST). A voucher specimen (VN-2406) was deposited at the Herbarium of the Institute of Ecology and Biological Resources of the Vietnam Academy of Science and Technology (VAST), Hanoi, Vietnam. The harvested fruits were carefully dried in a confined space at 40°C for 48 hours. The glandular trichomes were then separated and collected for further investigations by gently hand-rubbing dried fruits on a sieve of stainless-steel mesh.

### 2.3. Extraction and Isolation

Dry glandular trichomes (200 g) were successively extracted with EtOH (5 × 0.5 L). The extracts were combined and concentrated under diminished pressure. The residue (24 g) was suspended in water (70 mL) and extracted successively with *n*-hexane and EtOAc. The *n*-hexane and EtOAc solutions were concentrated under reduced pressure to afford 4.9 g and 10.5 g, respectively. The water solution was concentrated under vacuum to give 7.1 g of dry extract.

The EtOAc extract (10.5 g) was subjected to silica gel column chromatography (CC) eluted with a solvent gradient of CH_2_Cl_2_/MeOH to yield 9 fractions (F1-F9). F4 (1.5 g) was subjected to a CC on silica gel, eluting with CH_2_Cl_2_/MeOH gradient to obtain 5 subfractions (F4.1-F4.5). Subfraction F4.3 (137 mg) was purified on a Sephadex LH-20 column (100% MeOH) to yield compound **4** (pale yellow solid, 10.1 mg, yield = 0.005%). Subfraction F4.4 (99.6 mg) was purified on a Sephadex LH-20 column (100% MeOH) and then repurified by CC on silica gel (CH_2_Cl_2_/MeOH gradient) to provide compound **3** (pale yellow solid, 17.3 mg, yield = 0.0087%) and compound **5** (pale yellow solid, 15.5 mg, yield = 0.0078%). Fraction F6 (2.3 g) was subjected to a CC on silica gel, eluting with CH_2_Cl_2_/MeOH gradient to obtain 7 subfractions (F6.1-F6.7). Subfraction F6.5 (57 mg) was purified on a Sephadex LH-20 column (100% MeOH) to provide compound **2** (pale yellow solid, 9.2 mg, yield = 0.0046%) and compound **1** (yellow powder, 4.4 mg, yield = 0.0022%). Fraction F7 (1.9 g) was subjected to a CC on silica gel, eluting with CH_2_Cl_2_/MeOH gradient to obtain 6 subfractions (F7.1-F7.6). Subfraction F7.4 (97 mg) was purified on a Sephadex LH-20 column (CH_2_Cl_2_/MeOH: 1.5/8.5) to yield compound **6** (pale yellow solid, 4.7 mg, yield = 0.0024%).

**Macatanarin D (1)**: yellow powder; [*α*]^29^_D_ + 33.9 (*c* 0.05, MeOH); FT-IR (KBr) *ν*_max_: 3414, 2928, 1725, 1656, 1610, 1475, 1374 1163, 1087, 967, 894, 745, 647; UV (MeOH) *λ*_max_ (log *ε*): 210 (2.6), 271 (1.25), 327 (1.15), 366 (1.20); ^1^H and ^13^C-NMR spectral data (see Table 1); HR-ESI MS: m/z 481.1864 [M+H]^+^ (calcd. for C_27_H_29_O_8_, 481.1862).

### 2.4. Cytotoxic Assay

The cytotoxicity assays were carried out in triplicate in 96-well microtiter plates against KB cell line (mouth epidermal carcinoma cells) and MCF-7 cell line (breast cancer cells). Cells were maintained in Dulbecco's DMEM medium, supplemented with 10% fetal calf serum, L-glutamine (2 mM), penicillin G (100 UI/mL), streptomycin (100 *μ*g/mL), and gentamicin (10 *μ*g/mL). Stock solutions of compounds were prepared in DMSO/H_2_O (1/9), and the cytotoxicity assays were carried out in 96-well microtiter plates against cancer or normal cells (3 × 10^3^ cells/mL) using a modification of the published method [14]. After 72 h incubation at 37°C in air/CO_2_ (95 : 5) with or without test compounds, cell growth was estimated by colorimetric measurement of living cells stained by neutral red. Optical density was determined at 540 nm with a Titertek Multiskan photometer. The IC_50_ value was defined as the concentration of the sample necessary to inhibit the cell growth to 50% of the control. Ellipticine was used as a reference compound.

## 3. Results and Discussion

Compound **1** was isolated as a yellow powder, and its molecular formula of C_27_H_28_O_8_ was established by HRESIMS at m/z 481.1864 [M+H]^+^ (calcd. for C_27_H_29_O_8_, 481.1862). The FT-IR showed absorption bands at *v*_max_ 3414, 1657, 1610, and 1475 cm^−1^ indicating the presence of hydroxy, *α*, *β*-unsaturated carbonyl and aromatic ring functionalities, respectively. The UV absorption maximum at 366, 327, and 271 nm was typical for a flavonol-type compound [15]. The presence of a substituted flavonol skeleton was suggested by the analysis of ^1^H and ^13^C-NMR spectroscopic data (Table 1). The NMR spectroscopic data of **1** were similar to those of macakurzin B, which has been previously isolated from *M. kurzii*, except for the presence of a prenyl, acetyl, and OH groups [6]. In the ^1^H-NMR spectrum, the presence of an ABX system at *δ*_H_ 6.92 (d, *J* = 8.5 Hz), 7.80 (dd, *J* = 2.5, 8.5 Hz), and 7.83 (d, *J* = 2.5 Hz) and a singlet proton at *δ*_H_ 6.45 was observed in the aromatic region. Additionally, the ^1^H-NMR data also exhibited an acetyl group at *δ*_H_ 1.88 (3H, s), and two isoprenoid units: a 3-methyl-2-butenyl group (*δ*_H_ 1.71 and 1.72 (each 3H, s), 5.31 (1H, t, *J* = 7.5 Hz), and 3.28 (2H, d, *J* = 7.5 Hz)), and a 2,2-dimethyl-3-hydroxy-dihydropyrano ring (*δ*_H_ 1.20 and 1.34 (each 3H, s), 3.67 (1H, dd, *J* = 5.5, 7.5 Hz), 2.77 (1H, dd, *J* = 5.5, 17.0 Hz), and 2.40 (1H, dd, *J* = 7.5, 17.0 Hz)). The analysis of ^13^C-NMR data and 2D HSQC spectrum of **1** revealed the presence of 27 carbons, corresponding to a flavonol derivative with one acetyl group and two isoprene moieties (Table 1).

**Figure 1:**
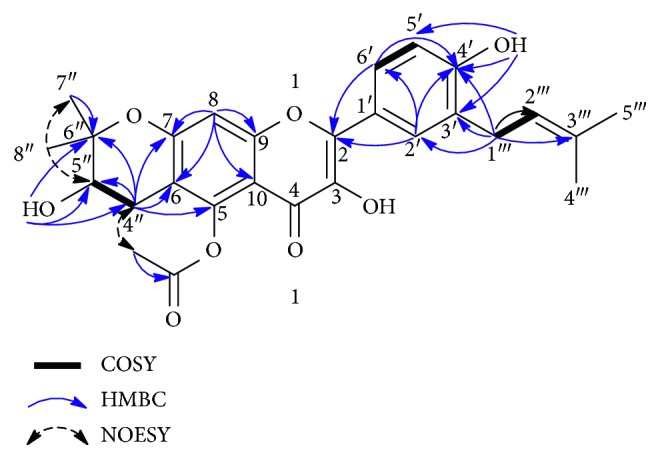
Key COSY, HMBC, and NOESY correlations for compounds **1**.

The HMBC correlations of H-1‴ (*δ*_H_ 3.28) with C-2′ (*δ*_C_ 128.2), C-3′ (*δ*_C_ 127.5), C-4′ (*δ*_C_ 156.1), C-2‴ (*δ*_C_ 122.6), C-3‴ (*δ*_C_ 131.6) and OH (*δ*_H_ 7.29) with C-4′ (*δ*_C_ 156.1), C-5′ (*δ*_C_ 114.9), and C-3′ (*δ*_C_ 127.5) determined the linkage of the isoprenyl chain with C-3′ and position of OH group at C-4′ on ring B (Figure 1). Furthermore, the COSY correlations of CH_2_-4″/H-5″ and the HMBC cross peaks of H-4″ (*δ*_H_ 2.40 and 2.77) with C-5″/C-6″/C-6/C-5/C-7 and OH (*δ*_H_ 3.09) with C-4″ (*δ*_C_ 26.0), C-5″ (*δ*_C_ 67.1), and C-6″ (*δ*_C_ 77.5) were confirmed that the position of OH group was at C-5″ of pyrano ring and 2,2-dimethyldihydropyranol moiety was attached to ring A. From the ^1^H-NMR spectrum, absence of resonance for a hydrogen-bonded hydroxy proton, led to the assumption that a free hydroxy group was not present at C-5. Two possible isomers could be considered at this stage. In the first one, the dihydropyranol moiety is fused to ring A via C-5 and C-6 and the *O*-acetyl group is located at C-7, while in the second possible isomer, the pyranol moiety is fused to ring A via C-6 and C-7 and the *O*-acetyl group is attached in position C-5. Since only the correlations of the methyl protons of acetyl group at *δ*_H_ 1.88 with methylene protons of CH_2_-4″ at *δ*_H_ 2.40 and 2.77 and no NOE interaction between the aromatic singlet H-8 (*δ*_H_ 6.45) and the methyl protons of acetyl group were observed on the NOESY spectrum, the pyranol moiety is supposed to be attached at C-6 and C-7 and the acetyl group in C-5 on ring A. This suggestion agreed with NMR data found for structurally close prenylated flavonoids. In particular, when a pyrane moiety is fused via C6 and C-7 on ring A, the resonance of C-7 is around *δ*_C_ 161.0–164.0 (*δ*_C_ 161.2 for dinklagin B [16] and 163.7 for tanariflavanone B [17], *δ*_C_ 161.1 for compound **1**), whereas a pyrane moiety fused via C5 and C-6 leads to a upfield-shifted carbon C-5 signal at about 155.0 (*δ*_C_ 155.5 and 155.7 for vogelins I and *J*, respectively [18]).

**Figure 2:**
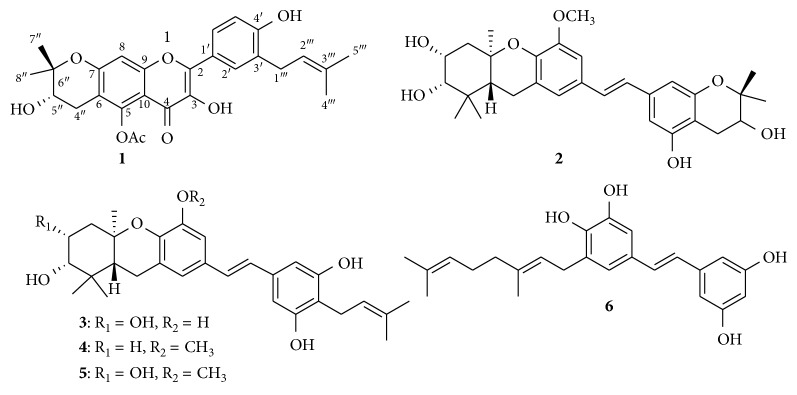
Structures of compounds **1–6** from fruit glandular trichomes of *M. tanarius*.

The relative configuration of C-5″ was established by proton coupling constant analysis and NOESY spectrum. The pseudoaxial orientation of H-5″ can be deduced from its proton coupling constants with a *gauche* (*J* = 5.5 Hz) and an *anti* (7.5 Hz). This observation was confirmed by the NOESY data analysis, which showed NOE correlations between H-5″ (*δ*_H_ 3.67) and CH_3_-7″ (*δ*_H_ 1.34) and proton H_b_-4″ (*δ*_H_ 2.77) of the 4″-CH_2_ group. Based on these observations, the structure of the newly isolated compound **1** was determined as 5-*O*-acetyl-6,7-(2,2-dimethyl-3-hydroxydihydropyrano)-3′-prenyl kaempferol (Figure 2) and named “macatanarin D.”

The structures of the known stilbenes: schweinfurthin H (**2**) [19], vedelianin (**3**) [20], schweinfurthin F (**4**), schweinfurthin E (**5**) [19], and 4′deprenyl-mappain (**6**) [21] were determined by analysis of spectroscopic data and comparison with reported data. So far, about 90% of the isolated compounds come from the leaves of *Macaranga* genus while 10% were isolated from other plant parts such stem and root barks, fruits, seeds, and flowers. No phytochemical studies had been conducted to date on glandular trichomes of *Macaranga* fruits. It is important to note that collecting time clearly influences the harvesting yield of glandular trichomes. While the young fruits do not have glandular trichomes and overripe fruits contain low yield of glandular trichomes, the adult/mature fruits, with clearly visible trichome glands, give the best results. In Vietnam, it is best to harvest mature fruits in June.

Since prenylated stilbenes and flavonoids of *Macaranga* genus are reported to have potent cytotoxic activities [9, 22], compounds **1**–**6** were evaluated for their cytotoxic activity against KB and MCF-7 human cancer cell lines. Ellipticine was used as a reference compound. The results are shown in Table 2. Compounds **1** and **6** showed moderate cytotoxic activities against KB cell line with IC_50_ values of 29.3 and 24.7 *μ*M, respectively. Compounds **3**, **4**, and **5** showed the strongest activities against both KB and MCF-7 cell lines with IC_50_ values in the range of 0.03–0.12 *μ*M, which is evenly stronger than ellipticine. It was worth noting that three aforementioned compounds possessed the same hexahydroxanthene moiety but a variable number of hydroxy groups, which may explain the difference in their cytotoxic potencies. Compound **2** also showed a significant cytotoxicity against KB cell line (IC_50_ = 0.26) but compared to compounds **3**, **4**, and **5**, cytotoxicity appears to be much less active against the MCF-7 cell line with an IC_50_ value of 10.4.

## 4. Conclusion

An undescribed flavonoid, macatanarin D (**1**), together with five known prenylated stilbenes (**2**–**6**) were isolated from glandular trichomes of fruits of *Macaranga tanarius*. Most of the compounds isolated have shown potent cytotoxic activities against the two cancer cell lines KB and MCF-7. It is postulated that these specialized metabolites are an important first line of defense against herbivorous insects and/or pathogens.

## Figures and Tables

**Table 1:** NMR data for compounds **1** in DMSO-d_6_.

Position	*δ*_C_^a^	*δ*_H_^b^ mult. (*J* in Hz)	Position	*δ*_C_^a^	*δ*_H_^b^ mult. (*J* in Hz)
2	141.3	—	4″	26.0	2.40 dd (7.5, 17.0)
					2.77 dd (5.5, 17.0)
3	136.7	—	5″	67.1	3.67 dd (5.5, 7.5)
4	170.7	—	6″	77.5	—
5	153.2	—	7″	25.5	1.34 s
6	104.4	—	8″	20.4	1.20 s
7	161.1	—	1‴	28.1	3.28 d (7.5)
8	93.5	6.45 s	2‴	122.6	5.31 d (7.5)
9	156.1	—	3‴	131.6	—
10	104.4	—	4‴	17.7	1.71 s
1′	122.0	—	5‴	25.5	1.72 s
2′	128.2	7.83 d (2.5)	C=O	172.0	—
3′	127.5	—	COCH_3_	21.5	1.88 s
4′	156.1	—	3-OH		6.74 s
5′	114.9	6.92 d (8.5)	4′-OH		7.29 s
6′	126.0	7.80 dd (2.5, 8.5)	5‴-OH		3.09 br s

^a^125 MHz; ^b^500 MHz. Assignments were made using the HSQC, HMBC, COSY, and NOESY spectra.

**Table 2:** Cytotoxic activities of compounds **1–6** against KB and MCF-7 cell lines.

Compounds	IC_50_ (*μ*M)	
	KB	MCF-7
**1**	29.3 ± 2.0	81.4 ± 3.9
**2**	0.26 ± 0.10	10.4 ± 1.0
**3**	0.050 ± 0.009	0.050 ± 0.006
**4**	0.10 ± 0.07	0.12 ± 0.05
**5**	0.050 ± 0.007	0.030 ± 0.009
**6**	24.7 ± 1.2	82.2 ± 3.7
**Ellipticine** ^a^	1.3 ± 0.2	2.4 ± 0.1

^a^Ellipticine was used as a positive control.

## Data Availability

The data used to support the findings of this study are available from the corresponding author upon request.

## Supplementary Materials

Supplementary Materials Figure 1S: HR-ESI-MS of 1. Figure 2S: ^1^H NMR spectrum of **1**. Figure 3S: ^13^C NMR spectrum of **1**. Figure 4S: HSQC spectrum of **1**. Figure 5S: HMBC spectrum of **1**. Figure 6S: COSY spectrum of **1**. Figure 7S: NOESY spectrum of **1**. ^1^H and ^13^C NMR spectroscopic data of isolated compounds **2**–**6**.
