# Supplementary material for: Cytotoxic Phenolic Compounds from Fruit Glandular Trichomes of Macaranga tanarius
Source: J Anal Methods Chem. 2019 Oct 13;2019:2917032. doi: 10.1155/2019/2917032 (PMC6815561; doi:10.1155/2019/2917032)
Supplement: Supplementary Materials — Figure 1S: HR-ESI-MS of 1. Figure 2S: 1H NMR spectrum of 1. Figure 3S: 13C NMR spectrum of 1. Figure 4S: HSQC spectrum of 1. Figure 5S: HMBC spectrum of 1. Figure 6S: COSY spectrum of 1. Figure 7S: NOESY spectrum of 1. 1H and 13C NMR spectroscopic data of isolated compounds 2–6. [file 2917032.f1.docx]

**SUPPORTING INFORMATION**

Cytotoxic Phenolic compounds from fruit glandular trichomes of *Macaranga tanarius*

Huong Doan Thi Mai,^1,2*^ Thuy Linh Nguyen,^1^ Thi Thanh Van Trinh,^1^ Van Nam Vu,^1^ Thi Dao Phi,^1^ Marc Litaudon,^3^ Fanny Roussi,^3^ Van Minh Chau,^1^ Van Cuong Pham^1,2*^

^a^Advanced Center for Bioorganic Chemistry, Institute of Marine Biochemistry, Vietnam Academy of Science and Technology, 18 Hoang Quoc Viet, Caugiay, Hanoi, Vietnam;

^b^*Graduate University of Science and Technology, VAST, 18 Hoang Quoc Viet, Caugiay, Hanoi, Vietnam*;

*^c^Institut de Chimie des Substances Naturelles, CNRS, ICSN UPR2301, University of Paris-Saclay, 91198, Gif-sur-Yvette, France*

^*^ Corresponding author:

Assoc. Prof. Dr. Huong Doan Thi Mai and Assoc. Prof. Dr. Van Cuong Pham

Institute of Marine Biochemistry

Vietnam Academy of Science and Technology

18 Hoang Quoc Viet, Caugiay, Hanoi, Vietnam

Tel.: 844 37564995

Fax: 844 38361283.

E-mail address: huongdm@imbc.vast.vn (Huong Doan Thi Mai); phamvc@imbc.vast.vn (Van Cuong Pham)

List of Content

[Figure 1S: HR-ESI-MS of 1 3](#_Toc18511210)

[Figure 2S: ^1^H NMR spectrum of 1 4](#_Toc18511211)

[Figure 3S: ^13^C NMR spectrum of 1 5](#_Toc18511212)

[Figure 4S: HSQC spectrum of 1 6](#_Toc18511213)

[Figure 5S: HMBC spectrum of 1 7](#_Toc18511214)

[Figure 6S: COSY spectrum of 1 8](#_Toc18511215)

[Figure 7S: NOESY spectrum of 1 9](#_Toc18511216)

[^1^H and ^13^C NMR spectroscopic data of isolated compounds 2‒6 10](#_Toc18511217)

# Figure 1S: HR-ESI-MS of 1


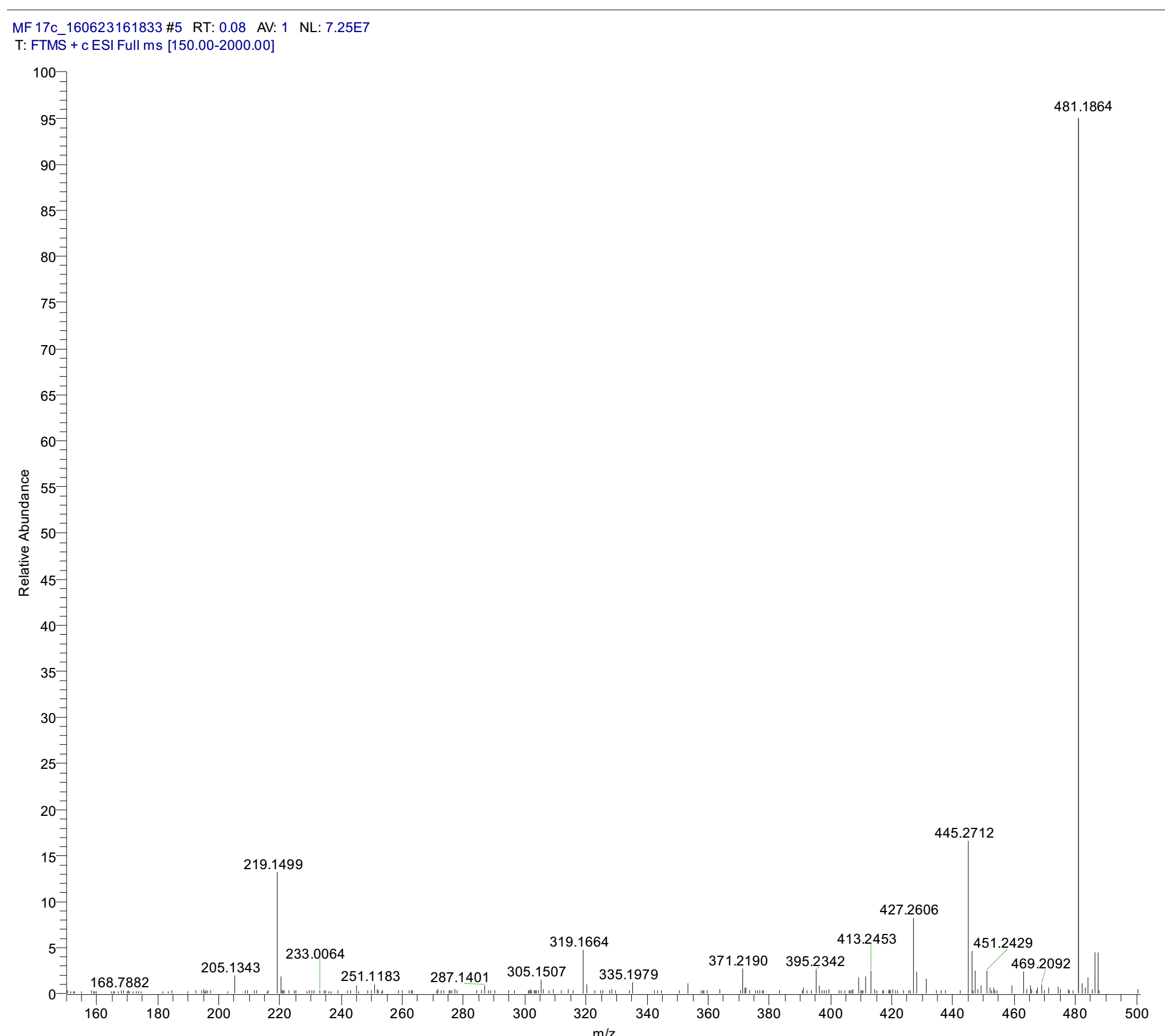

# Figure 2S: ^1^H NMR spectrum of 1


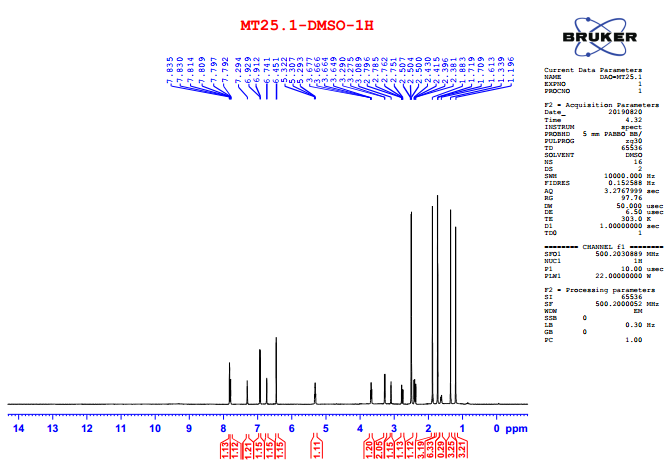

# Figure 3S: ^13^C NMR spectrum of 1


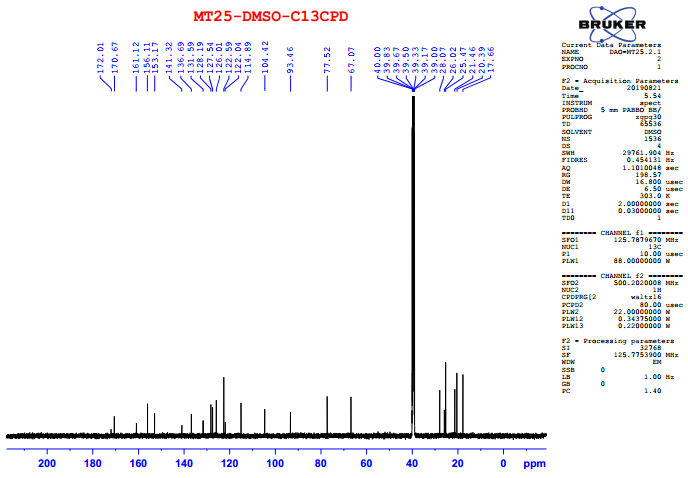

# Figure 4S: HSQC spectrum of 1


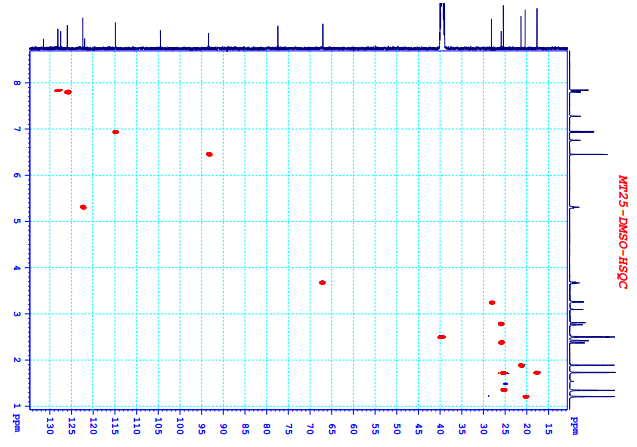

# Figure 5S: HMBC spectrum of 1


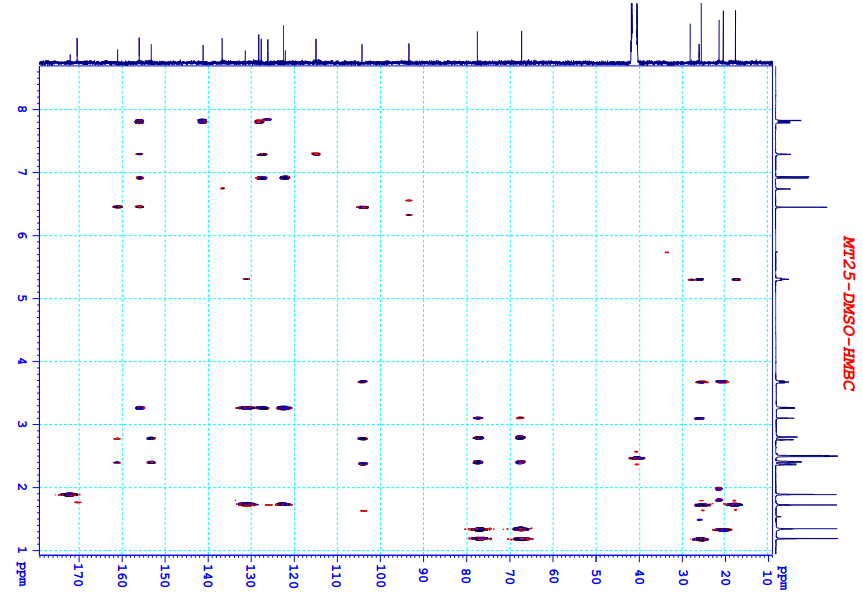

# Figure 6S: COSY spectrum of 1


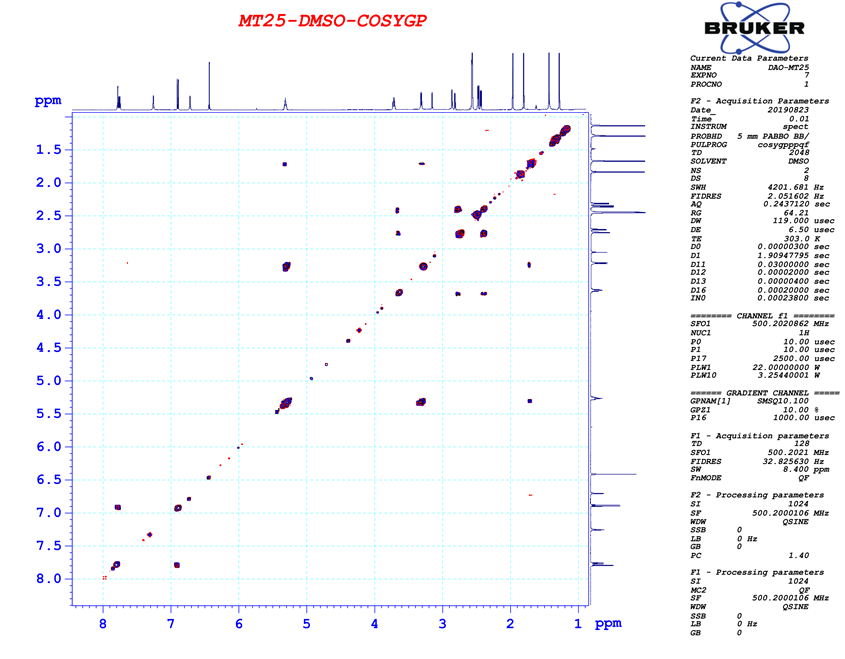


# Figure 7S: NOESY spectrum of 1


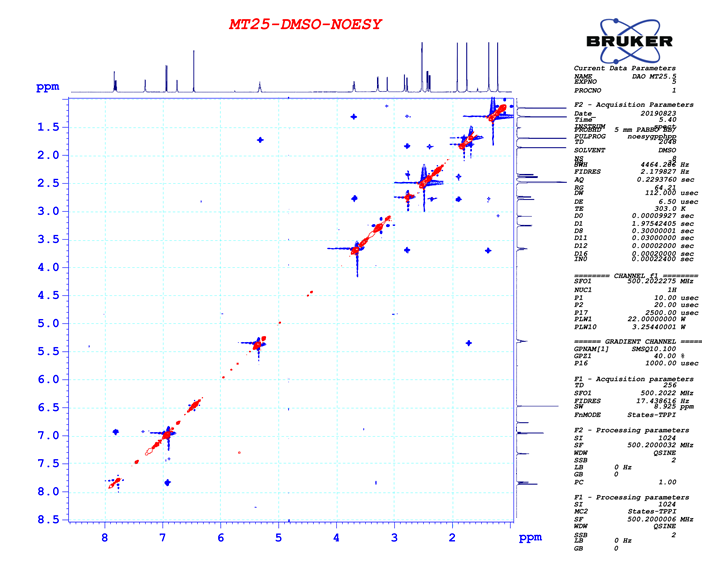

# **^1^H and ^13^C NMR spectroscopic data of isolated compounds 2**‒**6**

Schweinfurthin H (**2**): Pale yellow solid; [α] ^29^*_D_* + 33.5 (*c* 0.05, MeOH); ESI-MS *m/z* 511 [M+H]^+^; ^1^H NMR (500 MHz, CD_3_OD): *δ*_H_ 1.11 (3H, s, H-12), 1.13 (3H, s, H-11), 1.26 (3H, s, H-4′′), 1.35 (3H, s, H-5′′), 1.43 (3H, s, H-13), 1.75 (1H, dd, *J*= 5.5, 12.0 Hz, H-9a), 1.94 (1H, dd, *J*= 3.0, 14.0 Hz, H_a_-4), 2.37 (1H, dd, *J*= 3.0, 14.0 Hz, H_b_-4), 2.56 (1H, dd, *J* = 7.0, 17.0 Hz, H-1′′), 2.77 (2H, m, H-9), 2.93 (1H, dd, *J* = 5.5, 17.0 Hz, H-1′′), 3.34 (1H, m, H-2), 3.76 (1H, dd, *J* = 5.5, 7.5 Hz, H-2′′), 3.87 (3H, s, OMe-5), 4.17 (1H, q, *J* = 3.5 Hz, H-3), 6.47 (1H, d, *J* = 1.5 Hz, H-8′), 6.55 (1H, d, *J* = 1.5 Hz, H-4′), 6.82 (1H, d, *J* = 16.5 Hz, H-2′), 6.87 (1H, br. s, H-8), 6.91 (1H, d, *J* = 16.5 Hz, H-1′), 6.94 (1H, br. s, H-6). ^13^C-NMR (125 MHz, CD_3_OD): *δ*_C_ 16.5 (C-11), 20.8 (C-4′′), 21.9 (C-13), 24.0 (C-9), 25.8 (C-5′′), 27.4 (C-1′′), 29.4 (C-12), 39.2 (C-1), 48.8 (C-4), 49.2 (C-9a), 56.6 (OMe-5), 70.6 (C-2′′), 71.8 (C-3), 77.7 (C-3′′), 78.1 (C-4a), 78.8 (C-2), 105.1 (C-8′), 107.6 (C-6′), 108.4 (C-6), 108.5 (C-4′), 121.9 (C-8), 124.4 (C-8a), 127.5(C-2′), 129.1 (C-1′), 130.6 (C-7), 138.6 (C-3′), 143.5 (C-10a), 150.2 (C-5), 155.3 (C-7′), 157.6 (C-5′).

Vedelianin (**3**): Pale yellow solid; [α] ^29^*_D_* + 39.3 (*c* 0.11, MeOH); ESI-MS *m/z* 323 [M+H]^+^; ^1^H-NMR (500 MHz, CD_3_OD): *δ*_H_ 1.11 (3H, s, H-11), 1.12 (3H, s, H-12), 1.44 (3H, s, H-13), 1.67 (3H, s, H-4′′), 1.78 (3H, s, H-5′′), 1.78 (1H, m, H-9a), 1.96 (1H, br. d, *J* = 14.0 Hz, H_a_-4), 2.38 (1H, dd, *J* = 3.0, 14.0 Hz, H_b_-4), 2.75 (2H, m, H-9), 3.32 (1H, m, H-2), 3.31 (2H, d, *J* = 7.5 Hz, H-1′′), 4.17 (1H, br. s, H-3), 5.26 (1H, t, *J* = 7.5 Hz, H-2′′), 6.48 (2H, s, H-4′, H-8′), 6.72 (1H, *J* = 16.0 Hz, H-2′), 6.74 (1H, br. s, H-8), 6.82 (1H, br. s, H-6), 6.83 (1H, d, *J* = 16.0 Hz, H-1′). ^13^C-NMR (125 MHz, CD_3_OD): *δ*_C_ 16.5 (C-11), 17.9 (C-5′′), 22.0 (C-13), 23.3 (C-1′′), 23.9 (C-9), 25.9 (C-4′′), 29.4 (C-12), 39.2 (C-1), 44.7 (C-4), 48.9 (C-9a), 71.8 (C-3), 78.1 (C-4a), 78.8 (C-2), 106.6 (C-4′, 8′), 111.1 (C-6), 115.9 (C-6′), 120.4 (C-8), 124.2 (C-8a), 124.6 (C-2′′), 127.4 (C-2′), 128.7 (C-1′), 130.9 (C-7), 131.1 (C-3′′), 137.5 (C-3′), 141.9 (C-10a), 147.0 (C-5), 157.2 (C-5′, 7′).

Schweinfurthin F (**4**): Pale yellow solid; [α] ^23^*_D_* + 51.1 (*c* 0.12, MeOH); ESI-MS *m/z* 479 [M+H]^+^; ^1^H-NMR (500 MHz, CDCl_3_): *δ*_H_ 0.89 (3H, s, H-11), 1.10 (3H, s, H-12), 1.25 (3H, s, H-13), 1.65 (1H, m, H-3), 1.70 (1H, dd, *J* = 8.0, 10.5 Hz, H-9a), 1.77 (3H, s, H-4′′), 1.83 (3H, s, H-5′′), 1.88 (2H, m, H-3, H_a_-4), 2.12 (1H, m, H_b_-4), 2.70 (2H, m, H-9), 3.42 (1H, d, *J* = 7.0 Hz, H-1′′), 3.45 (1H, m, H-2), 3.88 (3H, s, OMe), 5.29 (1H, t, *J* = 7.0 Hz, H-2′′), 6.55 (2H, s, H-4′, H-8′), 6.82 (1H, s, H-8), 6.84 (1H, d, *J* = 16.0 Hz, H-1′), 6.85 (1H, s, H-6), 6.76 (1H, *J* = 16.0 Hz, H-2′), 6.88 (1H, *J* = 16.0 Hz, H-1′). ^13^C-NMR (125 MHz, CDCl_3_): *δ*_C_ 14.2 (C-11), 17.7 (C-5′′), 19.8 (C-13), 22.6 (C-1′′), 23.2 (C-9), 25.8 (C-4′′), 27.3 (C-12), 28.3 (C-3), 37.6 (C-4), 38.4 (C-1), 46.8 (C-9a), 56.1 (OMe), 77.0 (C-4a), 78.1 (C-2), 106.1 (C-4′, 8′), 107.2 (C-6), 112.9 (C-6′), 120.7 (C-8), 121.6 (C-2′′), 122.7 (C-8a), 125.8 (C-2′), 128.6 (C-1′), 128.9 (C-7), 135.2 (C-3′′), 137.2 (C-3′), 142.7 (C-10a), 148.9 (C-5), 155.2 (C-5′, 7′).

Schweinfurthin E (**5**): Pale yellow solid; [α] ^23^*_D_* + 49.2 (c 0.13, MeOH); ESI-MS *m/z* 495 [M+H]^+^; ^1^H-NMR (500 MHz, DMSO): *δ*_H_ 0.98 (3H, s, H-11), 1.00 (3H, s, H-12), 1.31 (3H, s, H-13), 1.61 (3H, s, H-4′′), 1.64 (1H, m, H-9a), 1.70 (3H, s, H-5′′), 1.83 (1H, m, H_a_-4), 2.16 (1H, dd, *J* = 3.0, 13.5 Hz, H_b_-4), 2.68 (2H, m, H-9), 3.17 (1H, m, H-2), 3.16 (2H, d, *J* = 7.0 Hz, H-1′′), 3.89 (3H, s, OMe), 3.99 (1H, m, H-3), 5.18 (1H, t, *J* = 7.0 Hz, H-2′′), 6.45 (2H, s, H-4′, H-8′), 6.76 (1H, *J* = 16.0 Hz, H-2′), 6.84 (1H, d, *J* = 16.0 Hz, H-1′), 6.87 (1H, s, H-8), 6.96 (1H, s, H-6). ^13^C-NMR (125 MHz, DMSO): *δ*_C_ 16.1 (C-11), 17.9 (C-4′′), 21.5 (C-13, C-1′′), 22.5 (C-9), 25.5 (C-5′′), 28.7 (C-12), 39.0 (C-1), 43.5 (C-4), 46.7 (C-9a), 55.6 (OMe-5), 69.7 (C-3), 76.6 (C-4a), 76.5 (C-2), 104.3 (C-4′, 8′), 107.3 (C-6), 115.9 (C-6′), 120.3 (C-8), 122.7 (C-8a), 123.5 (C-2′′), 126.3 (C-1′), 127.1 (C-2′), 129.3 (C-3′′), 130.9 (C-7), 135.5 (C-3′), 142.0 (C-10a), 148.6 (C-5), 156.0 (C-5′, 7′).

4’deprenyl-mappain (**6)**: Pale yellow solid; ESI-MS *m/z* 381 [M+H]^+^; ^1^H-NMR (500 MHz, CD_3_OD): *δ*_H_ 1.61 (1H, s, H-8′′), 1.67 (1H, s, H-9′′), 1.75 (1H, s, H-10′′), 2.07 (1H, q, *J* = 7.0 Hz, H-4′′), 2.14 (1H, q, *J* = 7.0 Hz, H-5′′),  3.32 (partially obscured by solvent, H-1′′), 5.14 (1H, br t,*J* = 7.0 Hz, H-6′′), 5.36 (1H, br t,*J* = 7.5 Hz, H-2′′), 6.17 (2H, t, *J* = 2.0 Hz, H-4′), 6.44 (2H, d, *J* = 2.0 Hz, H-2′, H-6′), 6.72 (1H, d, *J* = 16.5 Hz, H-β), 6.73 (1H, d, *J* = 2.0 Hz, H-2), 6.86 (1H, d, *J* = 2.0 Hz, H-6), 6.87 (1H, d, *J* = 16.5 Hz, H-α). ^13^C-NMR (125 MHz, CD_3_OD): *δ*_C_ 16.2 (C-10′′), 17.8 (C-8′′), 25.9 (C-9′′), 27.7 (C-5′′), 29.0 (C-1′′), 40.9 (C-4′′), 102.6 (C-4′), 105.8 (C-2′, 6′), 111.1 (C-6), 120.8 (C-2), 124.1 (C-2′′), 125.4 (C-6′′), 126.7 (C-β), 129.6 (C-3), 130.1 (C-α), 130.2 (C-1′), 132.2 (C-7′′), 136.7 (C-3′′), 141.4 (C-1), 144.4 (C-4), 146.1 (C-5), 159.6 (C-3′, 5′).
